# Tuberculosis — United States, 2018

**DOI:** 10.15585/mmwr.mm6811a2

**Published:** 2019-03-22

**Authors:** Amish Talwar, Clarisse A. Tsang, Sandy F. Price, Robert H. Pratt, William L. Walker, Kristine M. Schmit, Adam J. Langer

**Affiliations:** ^1^Division of Tuberculosis Elimination, National Center for HIV/AIDS, Viral Hepatitis, STD, and TB Prevention, CDC; ^2^Epidemic Intelligence Service, CDC.

In 2018, a total of 9,029 new tuberculosis (TB) cases were reported in the United States, representing a 0.7% decrease from 2017.* The U.S. TB incidence in 2018 (2.8 per 100,000 persons) represented a 1.3% decrease from 2017; the rate among non–U.S.-born persons was >14 times that in U.S.-born persons. This report summarizes provisional TB surveillance data reported to CDC’s National Tuberculosis Surveillance System (NTSS) through 2018. Although the total number of cases and incidence are the lowest ever reported in the United States, a recent model predicted that the U.S. TB elimination goal (annual incidence of <1 case per 1 million persons) will not be attained in the 21st century without greatly increased investment in detection and treatment of latent TB infection (LTBI) (*1*). Programs to identify, test, and treat populations at high risk for TB remain important to eliminating TB in the United States.

Health departments in the 50 states and District of Columbia (DC) electronically report provisional case data that meet the national TB surveillance case definition to CDC.^†^ Data reported include demographic information (e.g., birth date, sex, self-reported race/ethnicity, and country of birth), clinical information (e.g., reason for TB evaluation, anatomic site of disease, test results, and therapy administered), and information on TB risk factors (e.g., human immunodeficiency virus [HIV] infection status, history of homelessness, and residence in a congregate setting). According to U.S. Census Bureau definitions, a “U.S.-born” person is classified as one born in the United States or a U.S. territory or born abroad to a U.S. citizen parent. Race/ethnicity data are collected and reported using federal classification standards; Hispanics/Latinos can be of any race, and all other reported race categories are non-Hispanic/Latino. CDC derived the denominators used to calculate national and state TB incidence from July 2018 U.S. Census Bureau population estimates (*2*) and the denominators used to calculate TB incidence by national origin and race/ethnicity from July 2018 Current Population Survey data (*3*). The number of reported TB cases and TB incidence (cases per 100,000 persons) for 2017 and 2018, as well as the percent changes from 2017 to 2018, were calculated for the 50 states and DC and for each U.S. Census Bureau division. The numbers of TB cases and TB incidence per 100,000 persons were calculated by national origin and race/ethnicity for 2015–2018.

TB incidence declined 1.3% from 2017 to 2018 and an average of 1.6% per year during the last 4 years (2014–2018), a slower pace of decline than the 4.7% annual decline during 2010–2014.^§^ State-specific TB incidence for 2018 ranged from 0.2 per 100,000 in Wyoming to 8.5 in Alaska, with a median rate of 1.9 (Table 1). Ten states (Alaska, California, Florida, Hawaii, Maryland, Massachusetts, Minnesota, New Jersey, New York, and Texas) and DC reported TB incidence above the national rate. As has been the case for over 2 decades, four states (California, Florida, New York, and Texas) accounted for approximately half of the reported cases of TB in the United States.

**TABLE 1:** Tuberculosis (TB) case counts and incidence with annual percent changes, by U.S. Census division and state/district — 50 states and the District of Columbia, 2017 and 2018

Census division/State	No. of reported TB cases*			TB incidence^†^		
	2017	2018	% Change	2017	2018	% Change^§^
**Division 1: New England**						
Connecticut	63	51	−19.0	1.8	1.4	−19.0
Maine	14	14	0.0	1.0	1.0	−0.2
Massachusetts	209	200	−4.3	3.0	2.9	−4.8
New Hampshire	19	12	−36.8	1.4	0.9	−37.2
Rhode Island	13	20	53.8	1.2	1.9	53.7
Vermont	3	5	66.7	0.5	0.8	66.2
**Total**	**321**	**302**	**−5.9**	**2.2**	**2.0**	**−6.2**
**Division 2: Middle Atlantic**						
New Jersey	283	290	2.5	3.2	3.3	2.2
New York	800	750	−6.3	4.1	3.8	−6.0
Pennsylvania	192	212	10.4	1.5	1.7	10.3
**Total**	**1,275**	**1,252**	**−1.8**	**3.1**	**3.0**	**−1.8**
**Division 3: East North Central**						
Illinois	335	319	−4.8	2.6	2.5	−4.4
Indiana	100	116	16.0	1.5	1.7	15.4
Michigan	133	109	−18.0	1.3	1.1	−18.2
Ohio	149	178	19.5	1.3	1.5	19.2
Wisconsin	49	49	0.0	0.8	0.8	−0.4
**Total**	**766**	**771**	**0.7**	**1.6**	**1.6**	**0.5**
**Division 4: West North Central**						
Iowa	47	49	4.3	1.5	1.6	3.8
Kansas	29	28	−3.4	1.0	1.0	−3.5
Minnesota	178	172	−3.4	3.2	3.1	−4.1
Missouri	87	82	−5.7	1.4	1.3	−6.0
Nebraska	21	27	28.6	1.1	1.4	27.8
North Dakota	14	13	−7.1	1.9	1.7	−7.7
South Dakota	14	12	−14.3	1.6	1.4	−15.2
**Total**	**390**	**383**	**−1.8**	**1.8**	**1.8**	**−2.3**
**Division 5: South Atlantic**						
Delaware	15	22	46.7	1.6	2.3	45.1
District of Columbia	36	36	0.0	5.2	5.1	−1.0
Florida	549	591	7.7	2.6	2.8	6.0
Georgia	293	273	−6.8	2.8	2.6	−7.8
Maryland	207	207	0.0	3.4	3.4	−0.3
North Carolina	213	196	−8.0	2.1	1.9	−9.0
South Carolina	101	86	−14.9	2.0	1.7	−15.9
Virginia	204	205	0.5	2.4	2.4	−0.1
West Virginia	16	7	−56.3	0.9	0.4	−56.0
**Total**	**1,634**	**1,623**	**−0.7**	**2.5**	**2.5**	**−1.7**
**Division 6: East South Central**						
Alabama	120	91	−24.2	2.5	1.9	−24.4
Kentucky	65	65	0.0	1.5	1.5	−0.3
Mississippi	52	80	53.8	1.7	2.7	54.0
Tennessee	127	140	10.2	1.9	2.1	9.2
**Total**	**364**	**376**	**3.3**	**1.9**	**2.0**	**2.8**
**Division 7: West South Central**						
Arkansas	85	79	−7.1	2.8	2.6	−7.4
Louisiana	141	105	−25.5	3.0	2.3	−25.4
Oklahoma	54	74	37.0	1.4	1.9	36.7
Texas	1,127	1,129	0.2	4.0	3.9	−1.1
**Total**	**1,407**	**1,387**	**−1.4**	**3.5**	**3.4**	**−2.4**
**Division 8: Mountain**						
Arizona	188	178	−5.3	2.7	2.5	−6.9
Colorado	84	64	−23.8	1.5	1.1	−24.9
Idaho	10	15	50.0	0.6	0.9	47.0
Montana	3	5	66.7	0.3	0.5	65.2
Nevada	80	69	−13.8	2.7	2.3	−15.5
New Mexico	37	41	10.8	1.8	2.0	10.7
Utah	29	18	−37.9	0.9	0.6	−39.1
Wyoming	2	1	−50.0	0.3	0.2	−49.9
**Total**	**433**	**391**	**−9.7**	**1.8**	**1.6**	**−11.1**
**Division 9: Pacific**						
Alaska	53	63	18.9	7.2	8.5	19.2
California	2,059	2,091	1.6	5.2	5.3	1.1
Hawaii	116	120	3.4	8.1	8.4	3.7
Oregon	69	81	17.4	1.7	1.9	16.2
Washington	207	189	−8.7	2.8	2.5	−10.0
**Total**	**2,504**	**2,544**	**1.6**	**4.7**	**4.8**	**1.0**
**United States**	**9,094**	**9,029**	**−0.7**	**2.8**	**2.8**	**−1.3**

* Case counts were based on data from the National Tuberculosis Surveillance System as of February 11, 2019.

^†^ Cases per 100,000 persons. TB incidence was calculated using denominators from U.S. Census Bureau midyear population estimates.

^§^ Percentage change in incidence was calculated using unrounded rate for 2017 and 2018.

Among the 9,029 TB cases reported in the United States in 2018, approximately two thirds (6,276 [69.5%]) occurred in non–U.S.-born persons, whereas 2,662 (29.5%) occurred in U.S.-born persons; 91 (1.0%) cases occurred in persons for whom no national origin was documented (Table 2). This distribution is similar to that in 2017, when 6,392 (70.3%) cases occurred in non–U.S.-born persons, 2,693 (29.6%) occurred in U.S.-born persons, and 9 (0.1%) occurred in persons for whom no national origin was documented. TB incidence among non–U.S.-born persons (14.2 cases per 100,000) decreased by 3.8% from 2017 to 2018, and the incidence among U.S.-born persons (1.0 cases per 100,000) decreased by 1.8% (Figure).^¶^

**TABLE 2:** Newly diagnosed tuberculosis (TB) case counts and incidence,* by national origin and race/ethnicity — United States, 2015–2018^†^

U.S. population group	No. of cases (incidence)			
	2015	2016	2017	2018
**U.S.-born^§^**				
Hispanic	660 (1.8)	603 (1.6)	591 (1.5)	582 (1.5)
White, non-Hispanic	984 (0.5)	910 (0.5)	797 (0.4)	801 (0.4)
Black, non-Hispanic	1,142 (3.3)	1,066 (3.0)	1,008 (2.9)	938 (2.6)
Asian	138 (2.1)	146 (2.1)	134 (1.9)	139 (1.9)
American Indian/Alaska Native	144 (7.0)	110 (5.1)	92 (3.8)	102 (4.0)
Native Hawaiian/Pacific Islander	42 (6.1)	31 (4.3)	46 (6.7)	42 (5.6)
Multiple or unknown race/Ethnicity	25 (—^¶^)	23 (—^¶^)	25 (—^¶^)	58 (—^¶^)
**Total U.S.-born**	**3,135 (1.1)**	**2,889 (1.0)**	**2,693 (1.0)**	**2,662 (1.0)**
**Non–U.S.-born**				
Hispanic	2,036 (10.4)	1,990 (10.1)	1,973 (10.0)	2,006 (10.1)
White, non-Hispanic	258 (3.4)	286 (3.8)	268 (3.5)	251 (3.1)
Black, non-Hispanic	858 (23.2)	914 (22.7)	901 (22.2)	829 (19.9)
Asian	3,157 (29.7)	3,051 (27.2)	3,126 (27.3)	2,993 (25.4)
American Indian/Alaska Native	1 (1.9)	1 (2.9)	2 (2.9)	3 (5.2)
Native Hawaiian/Pacific Islander	60 (18.6)	47 (13.0)	66 (22.4)	74 (25.0)
Multiple or unknown race/Ethnicity	37 (—^¶^)	68 (—^¶^)	56 (—^¶^)	120 (—^¶^)
**Total non–U.S.-born**	**6,407 (15.3)**	**6,357 (14.7)**	**6,392 (14.7)**	**6,276 (14.2)**
Unknown national origin	5 (—^¶^)	7 (—^¶^)	9 (—^¶^)	91 (—^¶^)
**Overall total**	**9,547 (3.0)**	**9,253 (2.9)**	**9,094 (2.8)**	**9,029 (2.8)**

* Incidence was calculated as cases per 100,000 persons.

^†^ Case counts were based on data from the National Tuberculosis Surveillance System as of February 11, 2019. The Current Population Survey (https://www.census.gov/programs-surveys/cps.html) provides the population denominators used to calculate TB incidence according to national origin and racial/ethnic group.

^§^ U.S.-born persons were born in the United States or U.S. territories (American Samoa, Commonwealth of the Northern Mariana Islands, Guam, Puerto Rico, and U.S. Virgin Islands) or born elsewhere to a U.S. citizen. Non–U.S.-born persons were born outside the United States (or the U.S. territories), and include those born in the sovereign freely associated states (Federated States of Micronesia, Marshall Islands, and Palau) (unless one or both parents were U.S. citizens).

^¶^ Incidence was not calculated for these categories.

**FIGURE:**
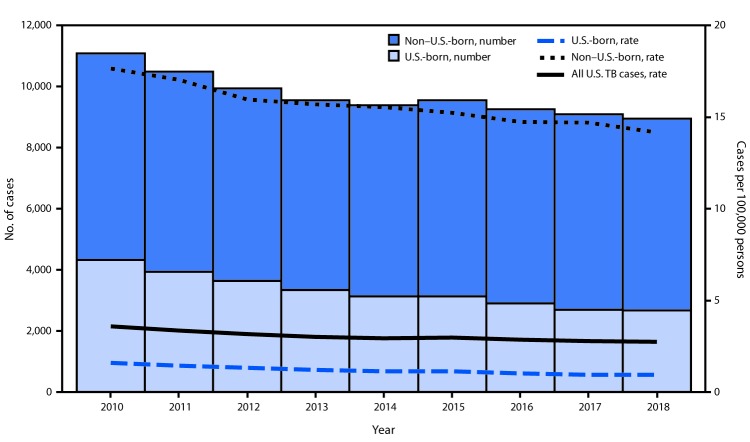
Number of tuberculosis (TB) cases and TB incidence, by national origin*^,†^ — United States, 2010–2018 * Number of cases among non–U.S.-born and U.S.-born persons and associated incidence exclude cases with unknown country of origin. Incidence for all U.S. TB cases includes cases with unknown country of origin. ^†^ Incidence for non–U.S.-born and U.S.-born persons calculated using population estimates from Current Population Survey. Incidence for all persons with TB diagnosed in the United States calculated using population estimates from U.S. Census Bureau.

Among non–U.S.-born persons with TB, incidence in 2018 was highest among Asians, followed by Native Hawaiians/Pacific Islanders, non-Hispanic blacks (blacks), Hispanics, and American Indian/Alaska Natives, and was lowest among non-Hispanic whites (whites) (Table 2). Among TB cases in non–U.S.-born persons, incidence decreased from 2017 to 2018 among Asians, blacks, and whites, but increased in Hispanics. The top five countries of birth of non–U.S.-born persons with TB were Mexico (1,195 cases; 19.0% of all non–U.S.-born cases), Philippines (781; 12.4%), India (616; 9.8%), Vietnam (503; 8.0%), and China (374; 6.0%). Among TB cases in non–U.S.-born persons, 2,905 (46.3%) were diagnosed ≥10 years after the patient first arrived in the United States.

The highest TB incidence for U.S.-born persons occurred among Native Hawaiians/Pacific Islanders, followed by American Indians/Alaska Natives, blacks, Asians, and Hispanics, and was lowest in whites (Table 2). Among U.S.-born persons, TB incidence decreased from 2017 to 2018 among blacks, but remained stable among Asians, Hispanics, and whites.

During 2018, 4.1% of TB cases were reported among persons who experienced homelessness within the year preceding diagnosis, 3.3% among residents of a correctional facility at the time of diagnosis, and 1.6% among residents of a long-term care facility at the time of diagnosis.** Among cases diagnosed in persons who experienced homelessness and among residents of long-term care facilities, 60.8% and 56.8%, respectively, were in persons who were U.S.-born, whereas among residents of a correctional facility, only 33.6% were U.S.-born. HIV status was known for 85.3% of TB cases reported in 2018. Overall, 5.3% of TB patients with known HIV status were coinfected with HIV, including 8.6% among persons aged 25–44 years.

Initial drug-susceptibility testing for at least isoniazid and rifampin was performed for 73.5% of all TB cases (and 93.8% of culture-confirmed cases) in 2017, the most recent year for which complete data are available.^††^ Among the 6,684 TB cases reported in 2017 with available drug-susceptibility testing results, 128 (1.9%) were multidrug-resistant TB.^§§^ Of these multidrug-resistant TB cases, 110 (85.9%) were in non–U.S.-born persons; 26 (20.3%) multidrug-resistant TB patients reported a previous episode of TB. Three cases of extensively drug-resistant TB^¶¶^ were reported, all of which occurred in non–U.S.-born persons.

## Discussion

In 2018, the provisional TB case count and incidence for the United States declined slightly, compared with those in 2017. Lower counts and incidences were seen in U.S.-born persons as well as in non–U.S.-born persons, who continue to represent a large majority of TB cases and have an incidence >14 times that of U.S.-born persons.

In 2018, approximately half (46.3%) of TB cases in non–U.S.-born persons received a TB diagnosis ≥10 years after first arriving in the United States, consistent with a published estimate that reactivation of remotely acquired LTBI has been responsible for >80% of domestic TB cases (*4*). Therefore, TB elimination will require a concerted effort to enhance surveillance, detection, and treatment for LTBI among populations at increased risk.

Between 3.1% and 5.0% of the U.S. population has LTBI (*5*,*6*). Without treatment, 5%–10% of persons with LTBI will develop TB disease in their lifetime (*7*). CDC and the U.S. Preventive Services Task Force recommend testing populations that are at increased risk for TB, including persons born in or who frequently travel to countries where TB is prevalent and persons who currently live, or previously lived, in congregate settings. CDC also recommends testing for TB in health care workers and others who work in places where there is a high risk of TB transmission, persons who are contacts of a person with infectious TB disease, and immunocompromised persons, who have a higher risk for developing TB disease once infected (*8*). According to one model, increased uptake of LTBI screening and treatment among populations at higher risk for TB would result in an incidence of 26 new infections per million by 2050 (*1*). Detection of LTBI can be improved by the preferential use of interferon-*γ* release assays over the tuberculin skin test, especially in persons with a history of Bacillus Calmette-Guérin vaccination or who are unlikely to return to have their tuberculin skin test read (*9*). In addition, the adoption of shorter, safer, and more convenient LTBI treatment regimens continues to be critical in improving treatment initiation and completion (*10*). Therefore, CDC recommends either 3 months of once-weekly rifapentine plus isoniazid or 4 months of daily rifampin for treatment of LTBI; these regimens may be used instead of longer courses of isoniazid alone (*10*). Given that the estimated prevalence of LTBI is higher among non–U.S.-born persons (*6*) and that rates of TB disease are much higher in this group, the detection and treatment of LTBI among non–U.S.-born persons should be prioritized. CDC is working with its state and local partners to develop an LTBI surveillance system to track effectiveness of public health measures to address LTBI.

The findings in this report are subject to at least two limitations. First, this analysis is limited to the reported provisional number of TB cases and incidence for 2018. Second, incidences are calculated using estimated population numbers as denominators.***

TB case counts and incidence in the United States in 2018 are the lowest ever reported, but this progress has slowed recently. To achieve TB elimination, the United States must expand detection and treatment of LTBI and TB disease. TB is a global problem, and its elimination will depend on cooperative measures to detect and treat LTBI and TB disease around the world.

**Summary**

**What is already known about this topic?**

The number of tuberculosis (TB) cases and incidence in the United States have steadily declined since 1993.

**What is added by this report?**

U.S. TB incidence in 2018 (2.8 cases per 100,000 persons) was the lowest ever reported. Non–U.S.-born persons accounted for approximately two thirds of cases.

**What are the implications for public health practice?**

The current decline in TB incidence is insufficient to eliminate TB in the United States in the 21st century. TB elimination will require enhanced surveillance, detection, and treatment. Focusing on populations that are at increased risk for latent TB infection will be important in achieving TB elimination.

## Footnotes

* This report is limited to National Tuberculosis Surveillance System provisional data as of February 11, 2019. Updated data will be available in CDC’s annual TB surveillance report later this year.

† https://www.cdc.gov/tb/programs/rvct/instructionmanual.pdf.

§ These calculations are based on unrounded annual TB incidence rates.

¶ The decrease in overall incidence does not fall within the range of decreases by national origin because, although the denominators used to calculate both rates increased from 2017 to 2018, the denominator used for rates by national origin (according to Current Population Survey data) increased by an additional 705,000 persons, compared with the denominator used to calculate the overall rate (according to U.S. Census Bureau data). This resulted in a larger calculated decrease in rate by national origin, compared with the overall rate.

** Percentages are calculated using cases with complete data for each of these three individual variables.

†† Because information on initial drug-susceptibility testing for isoniazid and rifampin is only available for 66.5% of all TB cases in 2018 (and 86.1% of culture-confirmed cases), more complete data from 2017 are presented instead. Culture-confirmed cases are defined as cases that were culture-positive on a specimen collected within 2 weeks of start of TB treatment.

§§ A case of TB caused by a strain of *Mycobacterium tuberculosis* that is resistant to at least isoniazid and rifampin.

¶¶ A case of TB caused by a strain of *Mycobacterium tuberculosis* that is resistant to isoniazid and rifampin as well as any fluoroquinolone and at least one of three injectable second-line drugs (i.e., amikacin, kanamycin, or capreomycin).

*** The second and third references provide information on population estimates used to calculate denominators.
